# Place identity as a mediator between motivation and tourist loyalty in ‘red tourism’

**DOI:** 10.1371/journal.pone.0284574

**Published:** 2023-10-27

**Authors:** Qiwen Dai, Shan Peng, Zijing Guo, Chunyu Zhang, Yanhong Dai, Wenjie Hao, Yanqiao Zheng, Wei Xu

**Affiliations:** 1 School of Economics and Management, Guangxi Normal University, Guilin, Guangxi, China; 2 Pearl River-Xijiang River Economic Belt Development Institute, Guangxi Normal University, Guilin, Guangxi, China; 3 School of Economics, Fujian Normal University, Fuzhou, Fujian, China; 4 School of Finance, Zhejiang University of Finance and Economics, Hangzhou, Zhejiang, China; 5 School of Culture and Communication, Guilin Tourism University, Guilin, Guangxi, China; Xiangtan University, CHINA

## Abstract

This paper constructs a theoretical analysis model based on the theories of planned behaviour, consumer emotion and identity by surveying tourists in Zunyi city, China and employing structural equation modelling to explore the influence mechanisms of tourist motivation, satisfaction and place identity on the loyalty of ‘red tourism’. The research results demonstrated a relationship between tourist motivation, satisfaction, place identity and tourist loyalty and thus confirmed the theoretical model. Tourist satisfaction and place identity are important means by which tourist motivation affects the loyalty of red tourism. However, tourist motivation cannot directly affect red tourism loyalty, though it can indirectly affect it via satisfaction and place identity. Tourist motivation not only directly influences the satisfaction of red tourism but also indirectly influences it through place identity. Furthermore, tourist motivation affects place identity. The mere recognition of place identity does not automatically attract tourists’ loyalty but can, through their experiencing satisfaction, indirectly inspire it. Nevertheless, place identity can only directly affect tourist satisfaction, and tourist satisfaction can only directly affect red tourism loyalty.

## Introduction

As a new form of tourism, Chinese red tourism is a political interpretation of heritage and a political socialisation campaign [1] launched in 2004 by the Chinese Communist Party (CCP). Red tourism is officially defined as a political project to consolidate and legitimise the CCP and to reinforce and enhance Chinese national identity [2]. ‘Red attractions’ denote state-sponsored nationalistic tourist sites, such as revolutionary memorials commemorating significant and heroic revolutionary figures and victorious events relating to the Red Army and the People’s Liberation Army, Chinese revolutionary-era war ruins, historical monuments of the CCP, residences of former communist leaders and other patriotic figures, as well various remnants of communist heritage [3, 4]. Since red tourism was implemented as a government socialist policy [1], its primary economic objects have been to promote people’s loyalty to the CCP’s leadership and improve economic development in traditional revolutionary base areas [3]. As a typical form of cultural, ideological and heritage tourism in China, it safeguards revolutionary heritage; offers opportunities to experience nostalgia; deepens national identity; teaches patriotism, history and culture and contributes to social realities, which has increasingly attracted attention from tourism scholars. Since the majority of revolutionary sites rich in red-tourism resources are situated in economically underdeveloped regions, various factors restrict the growth of red tourism, and potential issues have emerged over time, such as the insufficient protection of cultural resources and inadequate research into red cultural connotations. This has led to low-quality red tourism and thus low revisit rates among tourists [5], which is not conducive to the cultivation of their loyalty and in turn gravely limits the sustainable development of red tourism. How to improve red tourists’ loyalty, develop high-quality red tourism and make full use of its functions are important practical problems facing red places of interest.

Customer loyalty is a source of value and revenue and guarantees competitive advantage [6]. There exist a great many published articles on tourist loyalty [7–9], as its driving mechanism has garnered extensive attention from scholars. Studies have shown that tourist motivation [10, 11], perceived value [12, 13] and destination attachment [14] affected their behavioural loyalty, while satisfaction [15, 16], tourist destination identification [17, 18], destination trust [19], destination image [20], customer participation [9, 21–26] and brand reputation [7, 8, 27] affected tourists’ attitudinal loyalty. However, existing research on tourist loyalty principally focuses on hotels [28, 29], leisure tourism [30], heritage tourism [31] and popular tourist destinations [32, 33] rather than red tourism. The majority of articles on the latter centre on conception [34], the current situation [35], spatial distribution [36, 37], planning, management and promotion [38–41]. With few exceptions, such research tends to be fragmented, atheoretical and superficial. As such, the impact mechanism of red tourism loyalty requires further exploration. Prior studies have largely adopted tourists’ subjective value combined with tourist motivation as a single antecedent variable and used satisfaction as a mediating variable to analyse the influencing factors of tourist loyalty [9, 31]. Although the relationship between tourism motivation and loyalty has been discussed [31, 42], this has only been conceptually or superficially, while the conceptual explanation and logical connection between the two has been little explored. For tourism that actively attracts participation, destination identity is considered to be important [43, 44]; nevertheless, literature regarding the influence of tourist destination identification on tourist loyalty is scant, particularly in studies of red tourism. More research is required that examines red tourism from a political science perspective [1]. The development of red tourism has become an important political measure in China [38], with the government anticipating that it will influence the political attitudes of its citizens and thus promote national integration and individuals’ party identity [1]. Tourism is becoming an increasingly promising and popular industry and largely represents a form of consumerism and individualism, which supports the regional economy, impacts positively on destination attractiveness and contributes to sustainable cultural development and social environment [45]. Meanwhile, within this context, red tourism (as a special form of spiritual tourism) clearly differs from other types of tourism. What is the loyalty mechanism at work with red tourists, and will red tourist motivation and special emotions affect this loyalty? Despite the importance and universality of heritage politicisation via tourism, this is insufficiently charted territory in academic research [39]. The lack of investigation into the peculiarity of red tourism and red tourists as well as the absence of any consideration of memory or identity in existing research prompted this study to comprehensively examine the influence mechanism in red tourists’ loyalty. It aims to answer the question of, ‘Does identity contribute to red tourism loyalty through reconstruction and representation of communist heritage sites and, if so, how?’.

This study incorporates three theories: planned behaviour, consumer emotion and identity. The latter is used to investigate the identity of tourist destinations along three dimensions–culture, ethnicity and nationality–and a theoretical analysis model is constructed to determine the influence of identity on the loyalty of tourists who visit red tourist sites. Without an in-depth analysis of tourism politics, the dynamics and associated impacts of tourism development cannot be well understood [39]. This study builds upon factors previously explored in the development of tourism and supporting studies and, in particular, broadens the scope of future research by measuring various levels of government power and identity, which enriches the literature on place identity and tourist loyalty. This paper has theoretical and practical significance for developing high-quality red tourism, enhancing tourists’ confidence in Chinese culture, carrying forward national spirit and patriotism and promoting the implementation of the rural revitalisation strategy. The remainder of this paper is organised as follows: the subsequent section is the Literature Review, the third is Materials and Methods, the fourth is the Results, finally, the Discussion details the limitations of this study.

## Literature review

### Tourist motivation and tourist loyalty

Loyalty broadly denotes the commitment to purchase a product or service again [29, 46, 47]. Loyal consumers represent a stable income source, being less price sensitive and displaying a greater willingness to pay; thus, developing customer loyalty is a crucial marketing strategy. Such customers are less expensive to and serve may additionally attract new clientele through informal channels [21, 42]. In academics, empirical research on tourist loyalty is carried out from the perspective of behavioural loyalty and attitudinal loyalty–that is, the psychological tendencies of tourists when making travel decisions [25, 48, 49]–and mainly comprises two dimensions: the revisit intention and recommendation intention [50]. Motivation is deemed to be a demand [51], with tourism motivation being a complex psychological state that compels individuals to make travel decisions [51, 52] prompted by their needs and has a direct or indirect impact on their loyalty.

Tourism motivation can be classified into two ‘forces’–i.e. people travel because they are pushed and pulled to do so by certain forces [10, 52]. ‘Push’ motivations can be seen as the desire to escape, relax, go on an adventure, interact socially, improve health and fitness and gain prestige. ‘Pull’ motivations, on the other hand, include the attractiveness of a destination, such as its natural scenery, recreational facilities, cultural attributes and retail outlets [10, 53]. Therefore, people travel because they are ‘pushed’ by internal, psychological forces and ‘pulled’ by external forces, such as destination attributes, to make decisions regarding tourism [10, 52, 54]. Tourism motivations were identified by Crompton [55] as falling into nine categories: escape from a perceived mundane environment, exploration and evaluation of self, relaxation, prestige, return, enhanced kinship, promotion of socialisation, innovation and education [51]. Although tourist motivation is recognised as an important driver of tourist loyalty, a consensus in the academy has not yet been reached regarding their relationship and the influence mechanisms of loyalty [51]. If expectations are met sufficiently, tourists will experience satisfaction and their loyalty will have increased after their visit [53]. Some studies have found whether there is a positive correlation between tourist motivation and loyalty [6, 30, 35, 51, 53]. By contrast, the interplay between extrinsic motivation and service loyalty was not supported in a study conducted by Suardana et al. [56]. Such inconsistent results and the absence of structural investigations of the aforementioned theoretical frameworks in the context of red tourism in prior studies are the motivations behind the current research. Based on the above analysis, the following hypothesis is proposed:

**H1**: Tourist motivation has a positive and significant influence on tourist loyalty.

### The mediating effect of satisfaction on tourist motivation and tourist loyalty

Satisfaction denotes ‘a positive reaction resulting from the favourable assessment of consumption experience’ [31]. The concept of tourist satisfaction is evolved from customer satisfaction [57, 58] and is the quantification of tourists’ subjective feelings about their psychological perceptions [47, 59, 60]. Satisfaction is considered a necessary precondition for the formation of loyalty [47, 61], and satisfaction with tourist activities determines whether visitors return or recommend the destination to others [58, 62, 63]. The relationship between tourist satisfaction and tourist motivation has been confirmed by many empirical studies [10, 64, 65]. In fact, Ross and Iso-Ahola [64] argued that tourism motivation can explain over 90% of tourists’ overall satisfaction with their destination. Moreover, a study of the satisfaction of festival tourists by Lee et al. [66] revealed that if tourists’ motivation is fulfilled, they will positively evaluate the tourism experience. Pratminingsih et al. [67] explored tourist behaviour and demonstrated that motivation not only affects satisfaction but also directly impacts revisit intentions. Further, Yoon and Uysal constructed a model of tourist motivation, satisfaction and loyalty [10] and verified that tourists’ push and pull motivations not only directly affect their level of satisfaction but also indirectly affect their destination loyalty through this satisfaction. On the basis of the foregoing analysis, the following hypotheses are proposed:

**H2**: Tourist motivation has a positive and significant influence on tourist satisfaction.
**H3**: Tourist satisfaction has a positive and significant influence on tourist loyalty.
**H4**: Tourist motivation indirectly affects tourist loyalty through satisfaction.

### The mediating effect of place identity on tourist motivation and tourist loyalty

Proshansky [68] describes place identity as ‘the dimensions of self that define the individual’s personal identity in relation to the physical environment through a complex pattern of conscious and unconscious ideas, beliefs, preferences, feelings, values, goals and behavioural tendencies and skills relevant to this environment’. The role of the tourist guide is a significant element of post-modern identity, as tourism itself has become a postmodern phenomenon [69]. Early research on the influence of identity on loyalty principally involved the disciplinary fields of philosophy, sociology and psychology [70] and focused on regional dimensions, such as destination identity [71] and community identity [42] as well as connotative dimensions, such as organisation identity, national identity, political party identity [72–74], ethnic identity and brand identity [27, 29]. According to the theory of consumer sentiment [75], different levels of consumers’ identity will influence their attitudes and behaviours. For instance, brand identity acts as a mediator between enterprise association and brand loyalty [76]. Lee et al. [77] argue that social identity behaves as a mediating variable and influences the relationship between intrinsic altruistic motivation and community engagement behaviour. Furthermore, team identity has a mediating role in motivation and team loyalty [78]. Rather’s [29] study demonstrates that customer brand identification is positively correlated with loyalty, commitment, satisfaction and trust. Identity theory is increasingly being applied to the discipline of tourism, with Ma and Li [79] claiming that destination identity is based on the tourist’s interdependence on ‘self and destination’. Tourism destination identity at various levels is not only affected by tourist motivation but also serves as a mediator between tourist motivation and tourist loyalty. As the key motivation for visiting tourist destinations of historical significance, tourists’ involvement has a sizeable direct impact on destination loyalty as well as an indirect impact on it through local identity [80]. Destination identification further acts as a partial mediator between destination social responsibility motivation and revisit intention [81]. Based on the above analysis, the following hypotheses are proposed:

**H5**: Tourist motivation has a positive and significant influence on place identity.
**H6**: Red tourism destination identity has a positive and significant influence on tourist loyalty.
**H7**: Tourist motivation indirectly affects tourist loyalty through place identity.
**H8**: Tourist motivation indirectly affects tourist satisfaction through place identity.

### Satisfaction as a mediator between place identity and tourist loyalty

As important antecedent variables, satisfaction and place identity have a significant effect on loyalty [82] and a complex relationship. Life satisfaction, an essential concept in positive psychology, has a significant impact on identity and predicts future behaviours [83]. In literature [84], positive links were observed between vocational identity achievement and life satisfaction; however, the presence of a life calling was not related to life satisfaction. Chatman [72] found that organisation identity and member identity were significant predictors of members’ satisfaction and future intent to remain with the organisation. In addition, national identity and ethnic identity can positively predict life satisfaction [73]. In the field of tourism, the relationship between identity and satisfaction has also been studied, with tourists’ destination identity displaying a significantly positive effect on their shopping satisfaction [85] and their cultural identity significantly and positively affecting their sense of life satisfaction [86]. Moreover, identification and satisfaction are mutually causal and act as intermediary variables, indirectly affecting loyalty. Satisfaction is an important mediating variable on the loyalty of different groups in relation to their identities [87]. Studies in tourism have further shown that destination identity generally has a significant positive effect on tourists’ willingness to revisit through the experience of satisfaction [88]; when tourists identify with a particular destination, they are more likely to be satisfied with its services and facilities, then as that satisfaction accumulates and reaches a critical level, they feel a certain loyalty and are more likely to express a willingness to revisit [47, 58]. Cultural identity can play an active role in satisfaction in intangible cultural heritage tourism, where a high level of satisfaction with tourists leads to a high level of loyalty [89]. The current study on the mediating effects of identity is primarily concerned with the field of psychology. For example, organisational identity is an important mediator between employees’ job satisfaction and performance [90], while occupational identity partially mediates the relationship between career vocation and employee engagement [84]. Nonetheless, related research on tourism remains comparatively weak, investigating phenomena such as whether tourists’ overall satisfaction with hotels acts both directly and indirectly on loyalty, with the latter being through local brand identity [29]. On the basis of the above analysis, the following hypotheses are proposed:

**H9**: Place identity has a positive and significant influence on tourist satisfaction.
**H10**: Place identity indirectly affects tourist loyalty through satisfaction.

**Fig 1:**
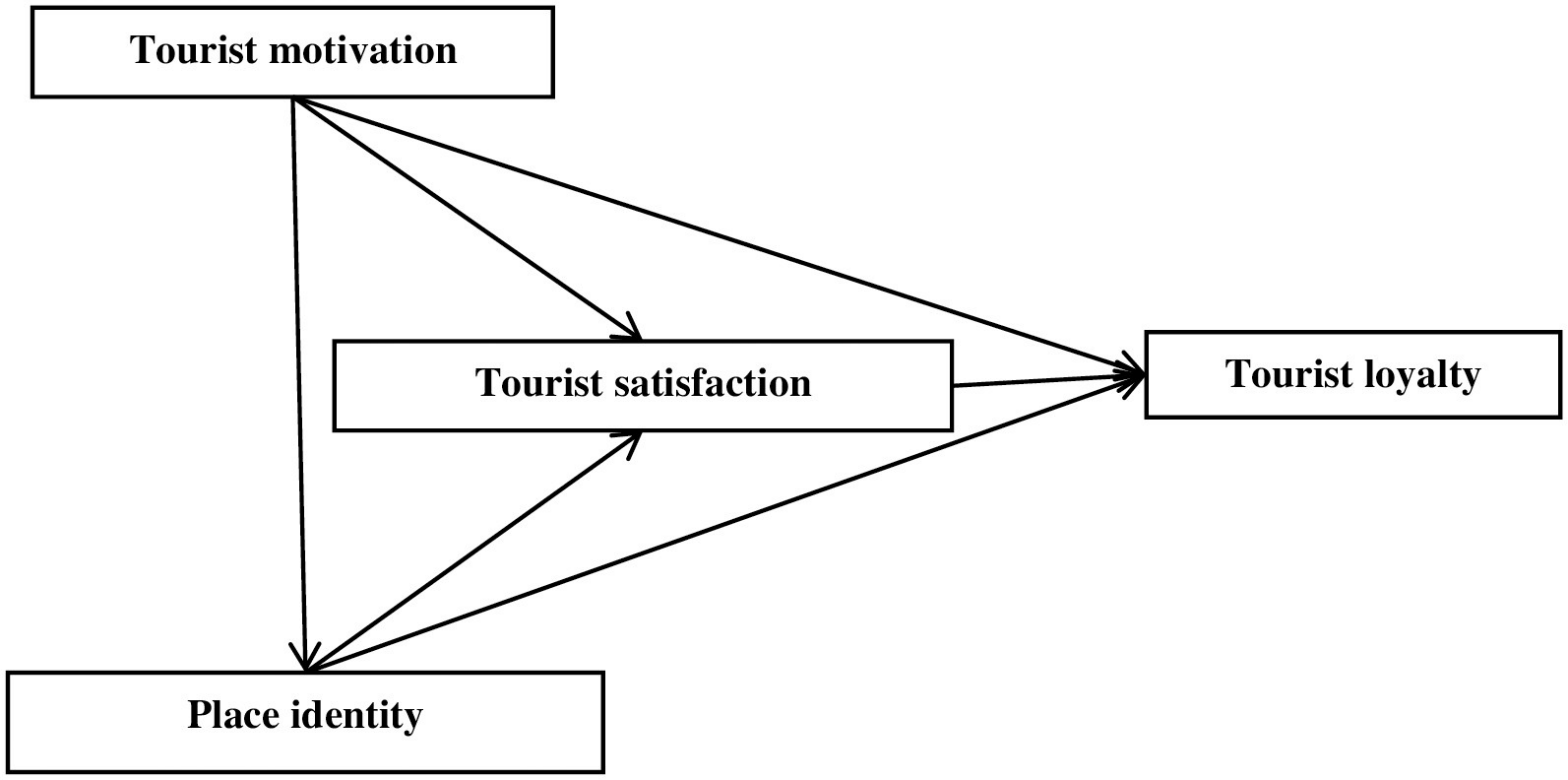
The conceptual method.

In order to test these hypotheses, this paper proposes to construct a conceptual model of the influence mechanism of tourist loyalty, as shown in Fig 1.

## Materials and methods

### Study area

The investigation was carried out in the city of Zunyi, which is located in the north of Guizhou province, western China. As one of the first city to be officially recognized as having historical and cultural significance in China and an excellent tourist destination, Zunyi was ranked 16th on the ‘2020 China Tourism Cities List’. Zunyi has one World Natural Heritage Site, one World Cultural Heritage Site, one national scenic spot, six national nature reserves, four national forest parks, one national geological park and 21 national AAAA tourist attractions. In 2019, Zunyi welcomed 193 million visitors and boasted a total tourism income of 21.63 billion yuan, an increase of 24.5% and 35.3% respectively. Zunyi is also China’s flagship city for red tourism, being one of the country’s leading red destinations and among the national culture and tourism consumption pilot cities. Zunyi has 518 famous revolutionary sites, such as the Zunyi Conference site, the former location of the Chishui River campaign, Loushan Pass, Wujiang River crossing and Hongjun mountain, and received the ‘2019 China National Tourism Best Red Tourism Destination’ award.

### Measures

The scales adopted in this study were taken from previous research, and the results of the tourist interviews in Zunyi were adapted and used for the dimensions in the initial model. To measure the variables, the 5-point/10-point Likert scale (1 = strong disagreement and 5/10 = strong acceptance) was selected. The model includes one exogenous and three endogenous latent variables. Tourist motivation was chosen as the exogenous latent variable and mainly comprised red tourism motivation and common tourism motivation, which were measured according to seven items adapted from a scale devised by Maghrifani et al. [51]. The endogenous latent variables included loyalty, satisfaction and tourism destination identification. Loyalty was measured against three items adapted from a scale proposed by Gallarza and Saura [91] and Oppermann [92]. Satisfaction consisted of total satisfaction and sub-satisfaction, with sub-satisfaction in turn being comprised of core attraction satisfaction and common attraction satisfaction; all of these were measured against 12 items based on the literatures of consumer behaviour [93]. Tourist destination identity was made up of cultural identity, ethnic identity and national identity and was measured according to six items adapted from a scale created by Rather, Su et al., Chatman and Jaspal et al. [29, 71–73]. The scale along with the items used and the descriptive statistics can be seen in Table 1.

**Table 1:** Descriptive statistics of the scale.

Latent variables	Items	Mean	SD
**Loyalty**	Revisit intention (L1)	0.910	0.281
	Recommend intention (L2)	0.920	0.265
	Share intention (L3)	0.860	0.345
**Satisfaction**			
**Overall satisfaction**	Total satisfaction (S1)	8.750	1.026
**Core attraction satisfaction**	Cultural atmosphere (S2)	8.830	1.004
	Red resources (S3)	8.910	1.023
	Participation (S4)	7.920	1.810
	Audio-visual effects (S5)	8.100	1.406
	Design (S6)	8.700	1.071
**Common attraction satisfaction**	Sanitation (S7)	8.950	0.956
	Convenience (S8)	6.870	2.578
	Security (S9)	8.890	0.999
	Price (S10)	8.160	1.359
	Commentary (S11)	8.140	1.571
	Services (S12)	8.690	1.335
**Tourist motivation**			
**‘Red tourism’ motivation**	Experience red culture (M1)	4.540	0.686
	Learn red spirit (M2)	4.010	0.867
**Common tourism motivations**	Sightseeing (M3)	4.180	0.988
	Relaxation (M4)	4.070	0.898
	Social communication (M5)	3.800	1.046
	Thirst for adventure (M6)	3.870	0.859
	Reputation (M7)	4.350	0.731
**Place identity**			
**Cultural Identity**	Red cultural cognition (I1)	4.460	0.702
	Red cultural confidence (I2)	4.340	0.622
	Red cultural education (I3)	4.650	0.579
	Revolutionary spirit (I4)	4.420	0.682
**Ethnic Identity**	National pride (I5)	4.400	0.660
**National Identity**	Path confidence of socialism with Chinese characteristics (I6)	4.300	0.642

### Sample and procedure

This study was conducted in accordance with the Declaration of Helsinki, and the Guangxi Normal University Institutional Review Board reviewed and approved the study protocol. All participants read and signed a consent form prior to taking part. The questionnaires completed on site are used for data collection. Due to the lack of a suitable sampling framework, convenient sampling was utilised to select the study respondents [24, 47]. Tourists in Zunyi city were selected as the survey subjects. In order to guarantee the representativeness of the sample, the city’s famous red scenic spots, such as the Zunyi Conference site, Loushan Pass, and Hongjun mountain, were selected. To minimise errors related to the measurement items, the investigator guided the participants throughout the process of completing the questionnaire, which afforded the opportunity of asking for further clarification on their responses to reinforce content validity. The survey took place during the summer months, between July and August 2020, when the number of tourists in the survey location was substantial. Our sample population was limited to tourists who had visited one or more red sites on at least one occasion. A total of 400 questionnaires were received, and 371 valid questionnaires were obtained after screening, with an effective rate of 92.75%.

In terms of the sample characteristics, the gender representation was reasonably balanced, with 52.8% males and 47.2% females. The following were the percentages within each age group: under 20 years old (15.9%), 20–30 years old (41.5%), 31–40 years old (21.0%), 41–50 years old (10.0%) and over 50 years old (11.6%). The majority of the participants (55.0%) held a degree, 7.8% were civil servants or military personnel and 20.2% were members of the Communist Party of China. Furthermore, 38.8% of the respondents lived outside Guizhou province, 29.1% were from ZunYi city, 36.1% and 24.0% had travelled for one day and two days respectively, while 39.9% had travelled for three days or more. In addition, 28.6% and 20.7% of tourists had experienced red tourism once and twice respectively, while 50.7% of tourists had travelled to red destinations three times or more.

## Results

Prior to analysis, it was necessary to first evaluate the measurement model–that is, to test the common method variance (CMV) to confirm its reliability and validity and the appropriateness of each measure index for explaining the model’s variables. Second, the structural model was analysed in terms of its goodness of fit and predictive ability, as well as the relationships (path coefficients) between the variables and their significance to judge the hypotheses proposed in the research framework.

### Common method variance

In accordance with Podsakoff et al. [94], we deployed Harman’s single-factor test to diagnose the potential influence of the CMV. The results demonstrated that the first factor explained just 16.25% (< 50%) of the total variance, indicating that the CMV in our data was limited.

### Reliability and validity of the model

The reliability and validity of the variables are shown in Table 2. As can be seen, the Cronbach’s Alpha values for tourist motivation, place identity and tourist satisfaction are equal to or greater than 0.6, indicating that the stability and reliability of the variables and overall scale are satisfactory. The Kaiser-Meyer-Olkin values for tourist motivation, place identity and tourist satisfaction are also greater than 0.6, and the Bartlett’s spherical test values were all statistically significant at the 1% level. The factor loadings of the majority of the variables are greater than 0.5, indicating good validity. Thus, the reliability and validity of the scale were verified, and the model was deemed appropriate for analysing the relationships between tourist motivation, satisfaction, place identity and tourist loyalty.

**Table 2:** Reliability and validity of the variables.

Variables	Satisfaction	Motivation	Place identity	Overall
**Number of items**	12	7	6	25
**Cronbach’s Alpha**	0.821	0.600	0.637	0.813
**KMO value**	0.810	0.625	0.665	0.775
**Bartlett’s spherical test value**	865. 466	206. 281	357. 358	1832. 069
**P**	0.000	0.000	0.000	0.000

### Confirmatory factor analysis

Prior to analysis, we processed the data using item parcelling, a technique widely utilized in social science research (e.g., Zhang et al. [95]). Initially, we calculated the mean of all items and used this as the unit of analysis. Next, we selected the item with the highest mean and the item with the lowest mean and combined them to form a new item.

The confirmatory factor analysis (CFA) was carried out using AMOS software to examine the construct validity. The hypothesised four-factor model yielded an acceptable fit, while alternative models demonstrated a poorer fit to the data (see Table 3).

**Table 3:** Confirmatory factor analysis.

Models	χ^2^/df	CFI	AGFI	GFI	TLI	IFI	RMR	RMESA
**Hypothesised four-factor model**	2.071***	0.896	0.909	0.934	0.872	0.898	0.033	0.054
**Three-factor model (combining TM and TS)**	3.141***	0.783	0.863	0.897	0.744	0.786	0.048	0.076
**Two-factor model (combining TL, TM and TS)**	3.184***	0.774	0.859	0.892	0.739	0.777	0.048	0.077
**Single-factor model (combining TL, TS, TM and PI)**	3.935***	0.693	0.831	0.870	0.649	0.698	0.050	0.089

Note: N = 371. Abbreviations: CFI = comparative Fit Index; AGFI = Adjusted Goodness of Fit Index; GFI = Goodness of Fit Index; df = degrees of freedom; IFI = incremental fit index; RMSEA = root-mean-square error of approximation; TLI = Tucker–Lewis index; χ^2^ = chi-square; RMR = root mean square residual; TL = Tourist loyalty; TS = Tourist satisfaction; TM = Tourist motivation; PI = place identity.

***p<0.001.

### Hypotheses test

Table 4 reveals that tourist motivation does not directly influence red tourist loyalty (β = 0.002, P>0.05), indicating that H1 is not accepted. Further, tourist motivation exerts a significant direct positive impact on satisfaction (β = 0.269, P<0.001). Thus, H2 is accepted. Meanwhile, satisfaction significantly and positively affects tourist loyalty (β = 0.541, P<0.001), and H3 is accepted. Tourist motivation has a significant direct effect on place identity (β = 0.218, P<0.05), which supports H5. Moreover, the effect of place identity on tourist loyalty is not significant (β = 0.015, P>0.05), indicating that H6 is rejected. Table 4 additionally shows that place identity directly influences satisfaction (β = 0.346, P<0.001). Thus, H9 is supported.

**Table 4:** Direct effect.

Structural relationship	Standardised coefficient	S. E.	C. R.	P
**Tourist motivation ⟶ Tourist loyalty**	0.002	0.036	0.020	0.984
**Tourist motivation ⟶ Tourist Satisfaction**	0.269	0.122	3.387	***
**Tourist satisfaction⟶ Tourist loyalty**	0.541	0.026	3.944	***
**Tourist motivation ⟶ Place identity**	0.218	0.050	2.361	0.018
**Place identity⟶ Tourist loyalty**	0.015	0.058	0.143	0.886
**Place identity⟶ Tourist satisfaction**	0.346	0.218	4.554	***

Notes:

***p<0.001.

Based on the mediation effect test procedure proposed by Preacher et al. [96], this study adopted the Bootstrap method using AMOS software.

Table 5 reveals that tourist motivation has an indirect effect on red tourist loyalty through satisfaction and place identity, with a mediating effect of 0.190; thus, H4 and H7 are confirmed. Because the direct impact of tourist motivation on loyalty is not significant, satisfaction and place identity are powerful intermediaries. However, tourist motivation indirectly affects satisfaction through place identity (β = 0.075, P<0.05), meaning H8 is supported. Thus, the direct, indirect and total effects of tourist motivation on satisfaction are all significant. The significant effect of satisfaction as a mediator between place identity and tourist loyalty (β = 0.187, P<0.01) reveals that H10 is support, and finally, owing to the direct effect of place identity on loyalty being insignificant, the mediating role of satisfaction is confirmed.

**Table 5:** Indirect effect.

	Pathway	Estimates	Bias-corrected 95-% Confidence Interval (BC)		Percentile 95-% Confidence Interval (PC)		P(BC/PC)
			Lower	Upper	Lower	Upper	
**Total effects**	Tourist motivation ⟶ Tourist loyalty	0.192	0.035	0.471	-0.003	0.409	0.058/0.124
	Tourist motivation ⟶Tourist satisfaction	0.344	0.164	0.490	0.185	0.514	*/*
	Place identity ⟶ Tourist loyalty	0.202	-0.029	0.465	-0.023	0.469	0.120/0.114
**Indirect effects**	Tourist motivation ⟶ Tourist loyalty	0.190	0.084	0.381	0.064	0.344	**/**
	Tourist motivation ⟶Tourist satisfaction	0.075	0.027	0.163	0.019	0.136	*/*
	Place identity ⟶ Tourist loyalty	0.187	0.096	0.386	0.060	0.304	**/**

Notes: ** *p*<0.01, * *p*<0.05.

## Discussion

### Theoretical contributions

Red tourism has attracted attention from the government, industry and academia. Nevertheless, few studies have investigated the loyalty of red tourists from the perspective of political science. Without an in-depth analysis of tourism politics, the dynamics of tourism development and its corresponding impacts cannot be comprehensively understood [39]. It is universally acknowledged that tourism contributes to social cohesion and patriotism [97] and thus may act as an agent in political socialisation. Chinese red tourism is officially a political project and serves as an ideological apparatus. Based on the theories of planned behaviour, consumption emotion and identity, a theoretical model of red tourism loyalty was constructed, focusing on the mediating role of tourism destination identity. This study selected Zunyi as the research area and adopted a questionnaire survey, structural equation model and intermediary-effect test as the methods to explore the correlation between tourism motivation, satisfaction, tourist destination identification and loyalty. Consequently, a complex direct and indirect relationship between these variables was revealed, which confirmed the theoretical model. The principal motivations for tourists participating in red tourism were learning red spirit and seeking experience. Additionally, both tourist satisfaction and destination identity were found to be important paths through which tourism motivation influences red tourism destination loyalty; in other words, tourism motivation does not directly influence red tourism loyalty, although it can have an indirect impact via satisfaction and destination identity. Satisfaction as a mediator between motivation and loyalty supported the empirical analysis provided by Yoon and Uysal [10]; however, the observed direct effect was inconsistent with the research by Yi et al, Yoon et al, He et al and Zhou et al. [6, 10, 53, 98], all of whom found that tourism motivation had a significant direct impact on customer loyalty, indicating that the influence of Tourist motivation on tourist loyalty varies among different types of trips. Although the motivation of pursuing novelty and experiencing the red spirit may be fulfilled in the initial trip, the marginal effect will be reduced; thus, encouraging tourists to revisit is a challenge. Furthermore, the contrast between tourist’s expectations and actual experiences of their chosen destinations is not conducive to the formation of loyalty.

Tourists’ core attraction satisfaction is predominantly influenced by audio-visual effects and red resources, while their common attraction satisfaction is mostly derived from commentary of tour guides and customer service, which implies that tourists favour the authentic experience of the red revolution spirit over the viewing experience. The results revealed that tourist motivation not only directly affects red tourism satisfaction but also indirectly impacts it through destination identity, which supports the empirical analyses by Yoon et al. and Ross et al. [10, 64] but is inconsistent with the study by Zhang et al. [99], which demonstrates that the role of tourist destination identity as a mediator between tourist motivation and satisfaction requires further analysis. The finding that tourist motivation directly affects red tourist destination identity corresponded with an empirical analysis by Zhang et al. [99], which also revealed a positive influence of visitors’ subject-related motivation on destination identity.

In accordance with Hung et al, Yoon et al and Tian et al. [10, 89, 100], this study found that tourist satisfaction can directly influence red tourism loyalty, while red tourism destination identity cannot, although it may exert an indirect influence through satisfaction. Conversely, Yi et al. [101] observed that exhibition identity had a direct impact on exhibition loyalty, and He et al. [102] noted significant direct and indirect effects of brand identity on the traditional antecedents of brand loyalty. Yang et al. [103] additionally concluded that the path from cultural identity to loyalty is through satisfaction. This study’s finding that red tourism destination identity can directly influence satisfaction backs up the empirical analysis by Yang et al. [103], which showed that each dimension of cultural identity exerts a positive influence on satisfaction. Upon experiencing the services and features of red tourism, visitors will form a positive perception and a certain degree of destination attachment, which will in turn enhance their satisfaction.

### Managerial implications

This paper offers some guidelines for marketers regarding red tourism destinations. First, it discloses that tourist satisfaction not only directly affects red tourism loyalty but that it is also an important means by which both tourist motivation and place identity indirectly affect it. These results provide a better understanding of how to develop tourists’ loyalty for managers of creative attraction businesses. Tourist companies and public institutions need to recognise that they can enhance tourists’ tourist motivation by enticing them to experience the red revolutionary spirit and to pursue novelty, which in turn will improve tourists’ satisfaction. These elements are important for generating continuous driving force and promoting tourists’ willingness to revisit, recommend or share red tourism destinations. Furthermore, to improve satisfaction with travel experiences, site managers must consider pull motivations, which relate to external sources, such as Zunyi’s red scenic spots. Tourists focus on authenticity and interpretation services at red destinations [104], and these variables have been shown to have the greatest direct impact on common attraction satisfaction in this paper. Therefore, an appealing presentation of the scenic spot and a high-quality service should be provided for tourists in order to maximise destination competitiveness. In addition, new technology can be adopted to improve the displays of red cultural relics and accompanying audio-visual effects, and the experience of participating in red cultural activities need to be strengthened, as these play a superior role in core attraction satisfaction.

Second, the results of this empirical research demonstrate that tourism destination identity affects tourists’ satisfaction and is also the important means by which motivation affects red tourism loyalty and tourists’ satisfaction. Therefore, when developing red tourism and cultural heritage tourism, it is essential to capitalise on the relationships between tourists’ identity and both loyalty and destination satisfaction and to fully promote these. Red tourism destination managers must focus particularly on training tour guides and other service personnel to effectively provide tourists with red revolutionary knowledge and culture and guide them to actively learn by translating the elements of red culture into special cultural symbols in the context of China’s core values to awaken tourists’ historical and cultural memory and help them construct cultural and national identities.

Third, empirical results have revealed that red cultural atmosphere has a significant effect on red tourist experiences [105] and is a core source of attraction and competitiveness in red tourism [106]. Businesses should take full advantage of Zunyi’s red resources, integrate natural scenery with red cultural elements and create a red cultural ambience using Chinese characteristics at red tourist sites. Tourists’ immersion in red-themed activities can enliven their perception of patriotism education and sense of red cultural identity [107]; for example, they can spontaneously channel the spirit of revolutionary heroes when tour guides relate the stories of generals and commanders [107]. These stories can inspire the design of various red-themed activities for tourists, enrich the diversity of Zunyi’s red tourism experience, help develop red culture and tourism with local characteristics and provide a foundation on which to build red tourist villages or towns to promote the high-quality development of red tourism.

### Limitations and future research

This research was not without its limitations. First, the proposed red-tourism-loyalty model was validated by tourists in Zunyi; however, the data, having been collected from this particular city, may not be applicable to other red tourist destinations with different characteristics, which may impair the model’s generalisability. Further research is therefore needed to improve the applicability and accuracy of the model through comparative analysis of studies of multiple areas using semi-structured interviews. Second, this paper analysed red tourists from a political standpoint, focusing on tourist destination identity. However, the tourist decision-making behavioural mechanism is intricate due to the complexity of tourists. For instance, different individuals possess different levels of knowledge, and views regarding heritage vary [108]; as such, perceptions and experiences of red tourism also vary [10, 109]. Some studies have examined the types of red tourists in China and have found them to have a range of motivations [104]. For example, historically motivated tourists are primarily interested in learning about the lives of great men and the revolutionary history of modern China, while others wish to educate themselves or their children. Thus, future research could analyse the loyalty mechanism behind red tourism from the perspective of different types of red tourists. Third, this article exclusively analysed the mediating roles of satisfaction and tourist destination identity, without considering the moderating effect of individual tourists. Some scholars have observed that gender and age moderate the impact of customers’ perceived value on their loyalty intentions [110]. Moreover, researchers have discovered that brand satisfaction significantly affects brand trust but to different degrees with each gender [111] and that well-educated guests are less loyal than poorly educated guests to the hotels they frequent [112]. Therefore, it is likely that views and emotions in relation to red tourism also vary between groups according to gender, age and level of education, and the moderating role of these elements in tourists must be considered in future. Fourth, red tourism aims to sustain the communist identity by developing a ‘socialist country of Chinese characteristics’ [4], but whether these goals have been achieved or not has yet to be analysed, which calls for future study. Similarly, the psychological and behavioural changes that take place in tourists after participating in red tourism, where they are exposed to phenomena such as hero worship and patriotism, requires investigation.
